# RETRACTION: The Influence of Minimally Invasive Esophagectomy on Wound Infection in Patients Undergoing Esophageal Cancer Surgery: A Meta‐Analysis

**DOI:** 10.1111/iwj.70385

**Published:** 2025-03-21

## Abstract

**RETRACTION:** GuoD. , LiaoF. , YangL. , LiuB. , and ChenL. , “The Influence of Minimally Invasive Esophagectomy on Wound Infection in Patients Undergoing Esophageal Cancer Surgery: A Meta‐Analysis,” International Wound Journal 21, no. 1 (2024): e14598, 10.1111/iwj.14598.38272810 PMC10789583

The above article, published online on 15 January 2024, in Wiley Online Library (http://onlinelibrary.wiley.com/), has been retracted by agreement between the journal Editor in Chief, Professor Keith Harding; and John Wiley & Sons Ltd. Following an investigation by the publisher, all parties have concluded that this article was accepted solely on the basis of a compromised peer review process. The editors have therefore decided to retract the article. The authors did not respond to our notice regarding the retraction.

**RETRACTION:** D. Guo , F. Liao , L. Yang , B. Liu , and L. Chen , “The Influence of Minimally Invasive Esophagectomy on Wound Infection in Patients Undergoing Esophageal Cancer Surgery: A Meta‐Analysis,” International Wound Journal 21, no. 1 (2024): e14598, 10.1111/iwj.14598.38272810 PMC10789583

The above article, published online on 15 January 2024, in Wiley Online Library (http://onlinelibrary.wiley.com/), has been retracted by agreement between the journal Editor in Chief, Professor Keith Harding; and John Wiley & Sons Ltd. Following an investigation by the publisher, all parties have concluded that this article was accepted solely on the basis of a compromised peer review process. The editors have therefore decided to retract the article. The authors did not respond to our notice regarding the retraction.

